# Increased level of acute phase reactants in patients infected with modern *Mycobacterium tuberculosis* genotypes in Mwanza, Tanzania

**DOI:** 10.1186/1471-2334-14-309

**Published:** 2014-06-05

**Authors:** Ruth Stavrum, George PrayGod, Nyagosya Range, Daniel Faurholt-Jepsen, Kidola Jeremiah, Maria Faurholt-Jepsen, Henrik Krarup, Martine G Aabye, John Changalucha, Henrik Friis, Aase B Andersen, Harleen MS Grewal

**Affiliations:** 1 Department of Clinical Science, Infection, Faculty of Medicine and Dentristry, University of Bergen, Bergen, Norway; 2 Mwanza Research Centre, National Institute for Medical Research, Mwanza, Tanzania; 3 Muhimbili Research Centre, National Institute for Medical Research, Dar Es Salaam, Tanzania; 4 Department of Human Nutrition, University of Copenhagen, Frederiksberg, Denmark; 5 Department of Clinical Biochemistry, Aalborg University Hospital, Aalborg, Denmark; 6 Clinical Research Centre, University of Copenhagen, Hvidovre Hospital, Hvidovre, Denmark; 7 Department of Infectious Diseases, Odense University Hospital, Odense, Denmark; 8 Department of Microbiology, Haukeland University Hospital, Bergen, Norway

## Abstract

**Background:**

There is increasing evidence to suggest that different *Mycobacterium tuberculosis* lineages cause variations in the clinical presentation of tuberculosis (TB). Certain *M. tuberculosis* genotypes/lineages have been shown to be more likely to cause active TB in human populations from a distinct genetic ancestry. This study describes the genetic biodiversity of *M. tuberculosis* genotypes in Mwanza city, Tanzania and the clinical presentation of the disease caused by isolates of different lineages.

**Methods:**

Two-hundred-fifty-two isolates from pulmonary TB patients in Mwanza, Tanzania were characterized by spoligotyping, and 45 isolates were further characterized by mycobacterium interspersed repetitive unit-variable number tandem repeat (MIRU-VNTR). The patients’ level of the acute phase reactants AGP, CRP and neutrophil counts, in addition to BMI, were measured and compared to the *M. tuberculosis* lineage of the infectious agent for each patient.

**Results:**

The most frequent genotype was ST59 (48 out of 248 [19.4%]), belonging to the Euro-American lineage LAM11_ZWE, followed by ST21 (CAS_KILI lineage [44 out of 248 [17.7%]). A low degree of diversity (15.7% [39 different ST’s out of 248 isolates]) of genotypes, in addition to a high level of mixed *M. tuberculosis* sub-populations among isolates with an unreported spoligotype pattern (10 out of 20 isolates [50.0%]) and isolates belonging to the ST53 lineage (13 out of 25 [52%]) was observed. Isolates of the ‘modern’ (TbD1-) Euro-American lineage induced higher levels of α_1_-acid glycoprotein (β = 0.4, *P* = 0.02; 95% CI [0.06-0.66]) and neutrophil counts (β = 0.9, *P* = 0.02; 95% CI [0.12-1.64]) and had lower BMI score (β = -1.0, *P* = 0.04; 95% CI[-1.89 – (-0.03)]). LAM11_ZWE (‘modern’) isolates induced higher levels of CRP (β = 24.4, *P* = 0.05; 95% CI[0.24-48.63]) and neutrophil counts (β = 0.9, *P* = 0.03; 95% CI[0.09-1.70]).

**Conclusion:**

The low diversity of genotypes may be explained by an evolutionary advantage of the most common lineages over other lineages combined with optimal conditions for transmission, such as overcrowding and inadequate ventilation. The induction of higher levels of acute phase reactants in patients infected by ‘modern’ lineage isolates compared to ‘ancient’ lineages may suggest increased virulence among ‘modern’ lineage isolates.

## Background

Genotyping of *Mycobacterium tuberculosis*, the causative agent of TB, may be utilized in order to study TB transmission dynamics, differentiate between possible outbreaks and laboratory cross-contamination, detect mixed infections and to evaluate the effectiveness of a TB control program by differentiating between relapse and re-infection. Although the members of the *M. tuberculosis* Complex show a high degree of sequence similarity at the genome level [1] the combination of different forms of genotyping such as Single Nucleotide Sequencing (SNP) and Large Sequence Polymorphisms (LSP) have shown that some lineages are associated with increased transmissibility, whereas others induce stronger host inflammatory responses [2]. The pathogenicity of an organism is determined by the organism’s virulence factors, which is described as the severity of disease caused by that organism, or the pathogens infectivity. Certain genotypes/lineages of *M. tuberculosis are* more likely to cause active TB in human populations from a distinct genetic ancestry [3,4] suggesting an adaptation by the pathogen to its human host. One lineage in particular, the W/Beijing lineage, has been studied extensively over many years due to its hypervirulence and increased level of multi-drug resistance. However, a recent study by Hanekom *et al.*[5] showed that this lineage in fact consists of 7 sub-lineages, where one sub-lineage shows increased resistance to anti-TB drugs, whereas another sub-lineage shows an increased level of transmissibility. This finding shows how differences among the infectious agents may impact their ability to cause infection and disease among their hosts.

TB has been shown to be associated with significant alterations in the levels of many serum proteins [6]. Acute phase reactants are proteins produce in the liver in response to an inflammation. The acute phase reactant C-reactive protein (CRP) is an established marker of acute inflammation [7], whereas the acute phase reactant α_1_-acid glycoprotein (AGP) has previously been associated with active TB [8] and latent TB [9].

Till date, there are no studies describing the distribution and frequency of the different genotypes of *M. tuberculosis* in Mwanza, Tanzania. As of 2012, the United Republic of Tanzania ranked 16 on the list of the 22 high-tuberculosis (TB) burden countries in the world [10]. Out of 909 cases which were tested in Tanzania for MDR-TB in 2009, 16 (1.8%) had MDR-TB, out of which 11 (68.8%) were new cases and 5 (31.2%) were previously treated patients [11]. If untreated, it has been shown that one smear-positive TB patient can infect up to 15 other people over the course of one year [12,13] highlighting the urgent need for rapid detection and adequate anti-TB treatment. Thus, the primary objective of this study was to genotypically characterize *M. tuberculosis* isolates collected from sputum smear-positive pulmonary TB patients in the city of Mwanza, using the well-established genotyping methods, spoligotyping and MIRU-VNTR. With the growing evidence that the genetic diversity among the *M. tuberculosis* genotypes/lineages may be an important factor with regards to clinical presentation, severity of disease and outcome of infection [14,15], the secondary objective included exploring differences in clinical presentation of TB disease and to determine whether any of these differences could be associated with the lineage of the infecting isolates.

## Methods

### Patient enrolment

This study was conducted in Mwanza city, in northwestern Tanzania. It is the second largest city in Tanzania. The city occupies the surface area of 1,325 sq kms, and according to 2002 census it had a population of about 500,000 [16]. Patients aged >15 years recruited at the local health facilities and confirmed as sputum culture positive at the Zonal TB Reference Laboratory were included in the study. Pregnant women, patients with terminal illness (from TB or any other serious disease unlikely to survive more than 48 h), non-residents (patients who would not stay in the study area for the entire period of 6 months of anti-TB treatment) and patients not willing to participate were not considered for enrolment. Patients diagnosed as sputum smear positive by microscopy using Ziehl-Neelsen (ZN) staining at the first visit to a diagnostic health facility were referred to any of the four main recruitment health facilities. Those who consented and for whom one or both spot and next day early morning sputum samples were ZN smear microscopy positive were requested to provide an additional early morning sputum specimen. Smear microscopy examination was performed using Auramine O staining and culture using Lowenstein-Jensen (LJ) solid media.

### Data collection

Following informed consent, information on the participants was collected using structured questionnaires at the first visit before initiation of anti-TB treatment. Venous blood (15 ml) was drawn from each participant for HIV testing, total lymphocyte count and CD4 cell counts. All *M. tuberculosis* isolates used in this study were collected from sputum smear positive pulmonary TB patients attending three of the major health clinics in Mwanza city from April 2006 –November 2008, according to WHO guidelines.

### Study sample

A total of 842 patients met the study criteria and were thus, included in this cohort. Information on data collection and laboratory procedures has been described in detail previously [17]. Serum concentrations of the acute phase reactants, C-reactive protein (CRP) and α_1_-acid glycoprotein (AGP) were determined at the Department of Clinical Biochemistry, Aalborg University Hospital, Denmark. Serum CRP was reported in mg/L and serum AGP in g/L with a lowest detection limit at 10 mg/L and 0.4 g/L, respectively. Serum levels of CRP >10 mg/L and AGP >1.2 g/L were reported as elevated. Weight and height were measured with the participant barefooted and with minimal clothing to the nearest 0.1 kg and 0.1 cm, respectively. The body mass index (BMI) was calculated as weight/height^2^ [kg/m^2^] [18]. Drug susceptibility testing (DST) was performed at the National Institute for Medical Research Laboratory, Mwanza, Tanzania for the 4 first line drugs isoniazid (H), rifampicin (R), streptomycin (SM) and ethambutol (E) according to the World Health Organization (WHO) recommendations.

### Ethics statement

Ethical permission was obtained from the Medical Research Coordinating committee of the National Institute for Medical Research (NIMR) in Tanzania, and consultative approval was given by The Danish National Committee on Biomedical Research Ethics. Written and oral information was presented to all eligible participants by the health staff before written informed consent was obtained. Written consent was obtained from parents/legal guardians of any participants under 18 years of age. Counseling prior to HIV-testing was compulsory, and post-test counseling was offered to all who tested HIV-positive. Participants with diagnosed HIV and/or diabetes were referred to the respective clinics for care and management.

### DNA isolation

Two-hundred-fifty-two *M. tuberculosis* isolates, which were available for this study, were sub-cultured on Löwenstein Jensen solid media at 37°C for 3–8 weeks and DNA was isolated using a boiling technique, where a 1 μl loop-full of bacterial cells were suspended in 200 μl of TE-buffer (10 mM Tris-Cl, 1 mM EDTA) and heat-killed by incubation at 95°C for 15–20 min [19]. The supernatant containing the DNA was collected by centrifugation at 12 000 rpm for 7 min. If heat extraction yielded DNA of poor quality causing difficulties for genotyping genomic DNA was isolated as previously described by van Embden [20].

### Spoligotyping

Spoligotyping was performed on genomic DNA by using the standard method as previously described [21]. Based on their spoligotype pattern, the isolates were assigned to families based on previously described criteria [22,23].

### MIRU-VNTR

Certain spoligotypes are more prone to be the product of admixing, thus creating a false spoligotype pattern [24]. A previous study conducted in South Africa [25] showed that 54% of isolates identified, by spoligotyping as belonging to the ST53 genotype, were comprised of mixed *M. tuberculosis* sub-populations. Thus, isolates belonging to the ST53 and ST451 (H37Rv) genotypes in addition to isolates with a previously unreported spoligopattern (according to the SITVIT database; http://www.pasteur-guadeloupe.fr:8081/SITVIT_ONLINE/), were chosen for MIRU-VNTR genotyping by amplification of 24 loci as described by Supply *et al*. [26] in order to rule out wrongful characterization due to mixed infections. Amplification was performed in a total volume of 20 μl containing 1 μl DNA, 0.04 to 0.4 μM of all 24 primer sets, and HotStart *Taq* Plus polymerase Master Mix (Qiagen). All reaction mixtures were subjected to 95°C for 5 min; 30 cycles of 30 s at 94°C, 1 min at 55°C, and 1.5 min at 72°C; and 7 min at 72°C. Analysis of the genotyping results was performed by multiplex PCR with a LIZ-1200 size standard (PE Applied Biosystems) for sizing of the PCR products. The PCR fragments were analyzed with a capillary-based electrophoresis sequencer (ABI 3730), and sizing of the various VNTR alleles was done with the Gene Mapper v/4.1 (PE Applied Biosystems). The number of repeats present at each locus was determined, and alleles were assigned numerical values accordingly.

Isolates with 100% identical MIRU-VNTR genotypes were defined as belonging to the same cluster.

#### ***Statistical analyses***

In exploratory analysis, linear and logistic regression models were used to test the association of *M. tuberculosis* lineages (Tables 1 and 2) with various background and clinical parameters. The models were adjusted for age, sex and HIV. Serum AGP was analyzed stratified by HIV status due to interactions (p < 0.05) between HIV and lineage groups with respect to AGP as a response. The data were assessed in three steps; comparing modern (TbD1-) against ancient (TbD1+) lineages, comparing EAI and Euro-American (Gagneux), respectively, against Indo-Oceanic (ancient), as well as comparing the most common modern spoligotypes, CAS_KILI and LAM11_ZWE, against the most common ancient spoligotype, EAI5. The models were compiled in Stata 12 (StataCorp LP, Texas).

**Table 1:** Background characteristics of 248 patients included in this study

**Variables**	**No. of patients (n = 248)**
**Age range (mean)**	15-81 (52)
**Gender**	
**Male**	150
**Female**	98
**Diabetes**	
**Yes**	6
**No**	241
**Unknown**	1
**HIV**	
**Positive**	95
**Negative**	153
**Previously treated**	
**Yes**	20
**No**	225
**Unknown**	3
**TB-contact**	
**Yes**	77
**No**	170
**Unknown**	1
**Smoking**	
**Yes**	53
**No**	195
**BCG scar**	
**Yes**	201
**No**	46
**Unknown**	1

**Table 2:** Overview of circulating lineages/genotypes in Mwanza, Tanzania and background characteristics of the infected patients

**Gagneux lineage**	**Brudey lineage**	**Shared-type**	**District**			**Sex**		**TB history**			**Previous TB treatment**			**HIV-infected**	
			**Buzuruga HC (n = 93)**	**Sekou Toure regional Hospital (n = 104)**	**Bugando medical centre (n = 51)**	**Female (n = 98)**	**Male (n = 150)**	**Unknown (n = 3)**	**New (n = 233)**	**Relapse (n = 12)**	**Unknown (n = 3)**	**No (n = 225)**	**Yes (20)**	**No (153)**	**Yes (95)**
**East African-Indian**	CAS	Unassigned	2	2	3	2	5	1	6	0	0	7	0	4	3
	CAS_KILI	ST21	17	16	11	18	26	0	42	2	0	40	4	25	19
	CAS1_DELHI	ST26	4	8	3	4	11	0	15	0	0	15	0	10	5
	CAS2	ST288	2	0	0	1	1	0	2	0	0	2	0	2	0
**East Asian**	BEIJING	ST1	0	1	2	2	1	0	3	0	0	2	1	2	1
**Euro-American**	F33	Unassigned	2	0	0	2	0	0	2	0	0	2	0	2	0
	H1	Unassigned	0	1	0	0	1	0	1	0	0	1	0	1	0
		ST218	4	3	0	4	3	0	7	0	0	5	2	5	2
	H3	ST50	1	2	1	3	1	0	4	0	0	4	0	2	2
	H37Rv	ST451	2	1	3	3	3	0	6	0	1	5	0	3	3
	LAM1	ST20	0	1	0	0	1	0	1	0	0	1	0	1	0
	LAM10_CAM	ST61	0	1	1	1	1	0	2	0	0	1	1	0	2
	LAM11_ZWE	ST1607	0	1	0	1	0	0	1	0	0	1	0	1	0
		ST1873	1	0	1	1	1	0	2	0	0	2	0	1	1
		ST59	21	19	8	25	23	0	45	3	1	44	3	32	16
	LAM4	ST811	1	0	1	1	1	0	2	0	0	2	0	1	1
	LAM8	Unassigned	1	0	0	0	1	0	1	0	0	1	0	0	1
	LAM9	ST42	2	4	0	2	4	0	6	0	0	5	1	4	2
	T1	Unassigned	3	3	0	1	5	0	6	0	0	6	0	5	1
		ST102	2	1	0	0	3	0	3	0	0	3	0	2	1
		ST1122	0	1	0	0	1	0	0	1	0	0	1	0	1
		ST373	0	2	0	0	2	0	2	0	0	1	1	0	2
		ST53	7	13	5	7	18	1	21	3	1	21	3	14	11
		ST612	0	1	0	1	0	0	1	0	0	1	0	1	0
	T2	ST135	0	1	1	1	1	0	2	0	0	2	0	1	1
		ST420	2	0	2	1	3	0	3	1	0	4	0	4	0
		ST52	0	0	1	0	1	0	1	0	0	1	0	0	1
	T3	ST37	1	3	0	1	3	1	3	0	0	4	0	4	0
	T3_ETH	ST149	0	2	0	2	0	0	2	0	0	2	0	0	2
	U	ST1570	0	0	1	1	0	0	0	1	0	0	1	0	1
**Indo-Oceanic**	EAI2	Unassigned	1	0	0	1	0	0	1	0	0	1	0	0	1
	EAI4	Unassigned	0	1	0	0	1	0	1	0	0	1	0	1	0
	EAI5	Unassigned	1	0	0	0	1	0	1	0	0	1	0	1	0
		ST126	12	11	6	6	23	0	29	0	0	28	1	17	12
		ST1369	1	1	0	1	1	0	2	0	0	2	0	2	0
		ST924	1	0	0	1	0	0	1	0	0	1	0	1	0
	EAI5 or EAI3	ST8	2	4	1	4	3	0	6	1	0	6	1	4	3

## Results

### Spoligotyping

Out of 252 isolates characterized by spoligotyping, 4 isolates gave a blank result whereas 248 isolates produced a spoligopattern. Background characteristics for the 248 isolates included in this study are presented in Table 1.

Out of these 248 isolates, 228 were assigned to 29 previously described shared-types [STs] [23] belonging to 4 previously defined lineages [23]. The Euro-American lineage (comprising the lineages as defined by Brudey [23] was the most common *M. tuberculosis* lineage. Out of 135 Euro-American lineage isolates, 125 isolates were assigned to 21 different STs (Table 2). The most frequent genotype was ST59 (n = 48 [19.4%]), belonging to the Euro-American lineage LAM11_ZWE, followed by ST21 (CAS_KILI lineage) comprising n = 44 out of 248 (17.7%) isolates (Table 2). Out of the 248 isolates which were subjected to spoligotyping, n = 20 (8.1%) isolates did not match with any of the previously described spoligo patterns [23] and thus, were not assigned to any of the previously defined STs (Table 2). The 20 isolates with previously un-described octal codes were assigned to lineages according to the web-based algorithm SPOTCLUST [27]. Out of 20 new isolates, 10 belonged to the Indo-Oceanic (CAS; n = 7 [35.0%], EAI2; n = 1 [5.0%], EAI4; n = 1 [5.0%], and EAI5; n = 1 [5.0%]) lineage and n = 10 isolates belonged to the Euro-American (T1; n = 6 [30%], F33; n = 2 [10.0%], LAM8; n = 1 [5.0%], and H1; n = 1 [5.0%]) lineage (Table 2). The spoligopattern and octal code for these 20 newly described isolates are given in Table 3.

**Table 3:** ***M. tuberculosis *** **isolates with previously unreported spoligopatterns identified in this study**

**ID**	**Octal code**	**Brudey lineage**^**1**^	**Gagneux lineage**^**2**^	**Mwanza urban district**	**MIRU-VNTR**
7	777777606063771	F33	Euro-American	Buzuruga HC	Mixed
149	777777747763771	F33	Euro-American	Buzuruga HC	Mixed
120	777777744020731	H1	Euro-American	Sekou Toure regional Hospital	Single
262	777777606061771	LAM8	Euro-American	Buzuruga HC	Mixed
122	747777777760730	T1	Euro-American	Sekou Toure regional Hospital	Single
160	777615647760771	T1	Euro-American	Buzuruga HC	Mixed
107	777737777760730	T1	Euro-American	Buzuruga HC	Single
224	777747777760771	T1	Euro-American	Sekou Toure regional Hospital	Single
183	777777477761771	T1	Euro-American	Buzuruga HC	Mixed
129	777777777721771	T1	Euro-American	Sekou Toure regional Hospital	Mixed
88	703377400001671	CAS	Indo-Oceanic	Buzuruga HC	Single
238	703377400003771	CAS	Indo-Oceanic	Sekou Toure regional Hospital	Single
26	703377740001771	CAS	Indo-Oceanic	Bugando Medical Centre	Mixed
202	703377740001771	CAS	Indo-Oceanic	Sekou Toure regional Hospital	Mixed
169	703777400003771	CAS	Indo-Oceanic	Bugando Medical Centre	Mixed
172	703777600000171	CAS	Indo-Oceanic	Bugando Medical Centre	Single
265	703777700000771	CAS	Indo-Oceanic	Buzuruga HC	Mixed
236	777000007413371	EAI2	Indo-Oceanic	Buzuruga HC	Single
29	777777740003231	EAI4	Indo-Oceanic	Sekou Toure regional Hospital	Single
132	777741757413771	EAI5	Indo-Oceanic	Buzuruga HC	Single

^1^ Lineages defined by Gagneux *et a*l. [23].

^2^ Lineages defined by Brudey *et al.*[22].

### MIRU-VNTR

Fifty-one isolates; 25 belonging to the ST53 genotype, and 20 isolates with previously unassigned spoligotypes were subjected to analysis by MIRU-VNTR for the purpose of identifying mixed *Mycobacterium* infections. Furthermore, as H37Rv is a laboratory strain, the presence of 6 isolates belonging to the ST451 (H37Rv) lineage was considered a laboratory contamination. This was confirmed by MIRU-VNTR. Out of the 45 ST53 and unreported spoligotypes analyzed by MIRU-VNTR, 22 samples consisted of a single *M. tuberculosis* sub-population (Table 4), whereas 10 out of the 20 (50%) unassigned isolates and 13 out of 25 (52%) ST53 genotypes were of mixed *Mycobacterium* sub-populations (i.e. harbored ≥2 alleles for ≥2 loci). The 24-loci MIRU-VNTR code for the 12 ST53 isolates and the 10 unassigned isolates, which were of a single sub-population, are provided in Table 4. Six isolates (samples 58 and 228; 38 and 46, and 94 and 242) formed 3 clusters (100% identical MIRU-VNTR code), comprising two isolates each (Table 4). There was no correlation between the occurrence of mixed infections and gender (odds ratio [OR], 0.55; 95% confidence interval [CI], 0.12-2.45; *P* = 0.29), HIV-status (OR, 0.77; 95% CI, 0.19-3.04, *P* = 0.45), or having a previous history of TB-infection (OR, 0.45; 95% CI, 0.15-7.24, *P* = 0.48).

**Table 4:** 24-loci MIRU-VNTR codes of isolates belonging to the ST53 genotype and isolates with previously unreported spoligopatterns

		**Mix 1**			**Mix 2**			**Mix 3**			**Mix 4**			**Mix 5**			**Mix 6**			**Mix 7**				**Mix 8**	
		**580**	**2996**	**802**	**960**	**1644**	**3192**	**424**	**577**	**2165**	**2401**	**3690**	**4156**	**2163b**	**1955**	**4052**	**154**	**2531**	**4348**	**2059**	**2687**	**3007**	**2347**	**2461**	**3171**
**Sample**	**Shared-type**	**MIRU 04**	**MIRU 026**	**MIRU 40**	**MIRU 10**	**MIRU 16**	**MIRU 31**	**VNTR 42**	**VNTR 43**	**AVNTR ETR-A**	**VNTR 47**	**VNTR 52**	**VNTR 53**	**VNTR QUB11b**	**VNTR 1955**	**VNTR QUB-26**	**MIRU 02**	**MIRU 23**	**MIRU 39**	**MIRU 20**	**MIRU 24**	**MIRU 27**	**VNTR 46**	**VNTR 48**	**VNTR 49**
21	ST53	2	5	2	3	4	3	2	4	2	2	3	*	4	2	4	2	5	2	2	1	3	4	2	3
38	ST53	2	1	2	4	1	2	1	4	3	4	3	4	2	3	7	2	5	2	1	1	3	4	2	3
46	ST53	2	1	2	4	1	2	1	4	3	4	3	4	2	3	7	2	5	2	1	1	3	4	2	3
56	ST53	2	1	2	4	1	3	1	4	3	4	3	4	2	3	7	2	5	2	2	1	3	4	2	3
58	ST53	2	1	2	4	1	2	1	4	3	2	3	4	2	3	7	2	5	2	1	1	3	4	2	3
94	ST53	2	5	4	3	1	3	2	4	3	2	3	3	4	2	*	2	3	2	2	1	3	3	2	3
136	ST53	2	5	*	3	1	3	2	4	2	4	7	3	2	2	*	2	5	2	2	1	3	4	3	3
166	ST53	2	1	2	*	1	3	1	4	3	4	4	4	1	3	*	2	5	2	2	1	3	4	2	3
228	ST53	2	1	2	4	1	2	1	4	3	4	3	4	2	3	7	2	5	2	1	1	3	4	2	3
237	ST53	2	1	4	5	1	3	4	2	3	2	3	*	2	3	3	2	5	2	2	1	3	4	2	3
242	ST53	2	5	4	3	1	3	2	4	3	4	3	*	4	2	2	2	3	2	2	1	3	3	2	3
249	ST53	2	1	2	4	1	3	1	4	3	2	3	*	2	3	5	2	5	2	2	1	3	4	2	3
29	Unassigned	2	2	4	4	4	5	1	2	9	2	5	2	9	6	6	2	6	3	2	2	3	3	4	3
88	Unassigned	2	1	4	7	4	4	4	2	4	2	2	*	2	3	4	2	5	3	2	1	3	4	2	3
107	Unassigned	2	4	3	3	3	4	2	5	4	2	3	3	2	3	7	2	5	2	1	1	3	4	1	0\3
120	Unassigned	2	4	3	3	3	4	2	5	4	3	4	4	2	3	7	2	1	2	2	1	3	4	1	3
122	Unassigned	2	5	3	3	3	3	2	3	3	2	3	3	2	3	7	2	5	2	1	1	3	2	2	3
132	Unassigned	2	2	2	4	2	5	2	4	7	4	2	2	7	8	5	2	6	2	2	2	3	3	5	3
172	Unassigned	2	1\7	4	4	6	6	4	2	4		2	4	2	4	4	2	5	1	2	1	2	4	2	3
224	Unassigned	2	5	4	1	2	3	2	4	3	2	3	3	4	3	5	2	5	2	1	1	3	4	3	3
236	Unassigned	4	2	3	4	3	4	2	4	9	2	4	2	6	6	5	2	4	3	2	2	3	3	3	3
238	Unassigned	2	1	4	7	4	4	4	2	3	2	2	*	2	3	5	2	5	3	2	1	3	4	2	3

Loci where a PCR product could not be produced are marked with ‘*’.

### *hsp65* sequencing

Four isolates which did not produce a spoligo pattern, were subjected to sequencing of the *hsp65* gene and compared to the NCBI database (BLASTN). One of the isolates showed a 100% match with the *Mycobacterium fortuitum* lineage, whereas the other 3 isolates matched 100% with the *M. tuberculosis* complex.

### Drug susceptibility testing (DST)

Out of the 248 isolates with a valid spoligotyping result DST was performed on 244 isolates. The DST showed that 231 isolates (94.7%) were sensitive to all four first-line anti-TB drugs, 13 were found to be resistant to any of the 4 first-line drugs (H: n = 7, SM: n = 2, H/SM: n = 2, H/SM/E: n = 1, H/E: n = 1), including 1 multi-drug resistant isolate (resistant to both rifampicin and isoniazid in addition to streptomycin and etambuthol). Nine of the 13 resistant isolates belonged the Euro American lineage (LAM11_ZWE [n = 4], T3 [n = 2], H1, [n = 1], LAM9 [n = 1], and H37Rv [n = 1]. Four isolates belonged to the East African (CAS_KILI [n = 4]) lineage. The 1 MDR isolates belonged to the EAI5/Euro American lineage.

### Comparison between groups/lineages

The logistic regression analyses showed that among HIV negative patients levels of AGP was higher (β = 0.3, *P* = 0.05; 95% CI [0.005 – 0.59]) in patients infected with a ‘modern’ (TbD1-) *M. tuberculosis* strain as compared to patients infected with isolates belonging to the ‘ancient’ (TbD1+) reference group (Table 5, left model).

**Table 5:** **Associations between infection by ‘ancient’ and ‘modern’ *M. tuberculosis *** **sublineages (according to Gagneux *et al.*** [22]**) and clinical manifestations**

**Groups**	**Reference group: ‘Ancient’ (n = 42)**			**Reference group: Indo-Oceanic (n = 42)**					
	**‘Modern’ (n = 183)**			**EAI (n = 64)**			**Euro-American (116)**		
**Variables**	**Regression coefficient**	**95% CI**	**P-value**	**Regression coefficient**	**95% CI**	**P-value**	**Regression coefficient**	**95% CI**	**P-value**
Age (years)^1^	-0.6	[-4.80 – 3.68]	0.80	1.0	[-3.89 – 5.93]	0.68	-1.4	[-5.84 – 3.11]	0.55
Weight (kg/m^2^)^2^	-2.0	[-1.63 – 0.16]	0.11	-1.7	[-4.82 – 1.42]	0.29	-2.4	[-5.25 – 0.44]	0.1
BMI (kg/m^2^)^2^	-0.7	[-1.63 – 0.16]	0.11	-0.5	[-1.52 – 0.52]	0.33	-1.0	[-1.89 - -0.03]	**0.05**
CRP (mg/L)^2^	14.2	[-4.20 – 32.52]	0.13	8.0	[-13.18 – 29.21]	0.46	18.5	[-0.85 – 37.76]	0.06
AGP (g/L)^1*^	0.3	[0.005 – 0.59]	**0.05**^3^	0.2	[-0.11 – 0.55]	0.19	0.4	[0.06 – 0.66]	**0.02**
Neutrophil count (x 10^9^ cells/L)^2^	0.6	[-0.13 – 1.32]	0.11	0.1	[-0.71 – 0.96]	0.77	0.9	[0.12 – 1.64]	**0.02**
Lymphocyte count (x 10^9^ cells/L)^2^	0.2	[-0.16 – 0.49]	0.32	0.3	[-0.08 – 0.67]	0.12	0.1	[-0.26 – 0.42]	0.65
CD4 count (cells/ul)^2^	-21.2	[-122.66 – 80.36]	0.68	21.9	[-95.47 – 139.35]	0.71	-45.9	[-152.95 – 61.19]	0.40
Hemoglobin (g/dl)^2^	0.03	[-0.68 – 0.75]	0.93	0.3	[-0.50 – 1.15]	0.44	-0.2	-0.91 – 0.59	0.67
Fasting blood glucose (mmol/L)^2^	0.1	[-0.32 – 0.51]	0.65	-0.02	[-0.48 – 0.45]	0.95	0.1	[1.71 – 5.08]	0.59
Diabetes (Yes)^2^	1.5	[0.55 – 4.24]	0.42	1.2^¶^	[0.35 – 3.75]	0.81	1.7^¶^	[0.60 – 4.97]	0.27
Smoking (Yes)^2^	2.6	[0.32 – 20.81]	0.38	1.5^¶^	[0.54 – 3.97]	0.46	0.9^¶^	[0.32 – 2.23]	0.74
TB contact (Yes)^2^	1.3	0.59 – 2.70	0.55	0.2^¶^	[0.50 – 2.90]	0.67	1.2^¶^	[0.56 – 2.76]	0.59
BCG scar^2^	1.5	[0.63 – 3.32]	0.39	1.1^¶^	[0.43 – 2.83]	0.83	1.7^¶^	[0.69 – 4.13]	0.25

Results are based on a linear and logistic regression models adjusted for age, sex and HIV status.

^1^Adjusted for HIV status and gender

^2^Adjusted for HIV status, age and gender

^3^*P*-values <0.05 are in bold.

*HIV negative only

^¶^Odds ratio

BMI: Body Mass Index.

EAI: East-African Indian.

CRP: C-reactive protein.

AGP: Orosomucoid.

BCG: Bacillus Calmette-Guerrin.

Patients infected with isolates belonging to the Euro-American lineage (‘modern’) had lower BMI score (β = -1.0, *P* = 0.04; 95% CI [-1.89 – (-0.03)]) in addition to higher levels of AGP (β = 0.4, *P* = 0.02; 95% CI [0.06-0.66]) and neutrophil count (β = 0.9, *P* = 0.02; 95% CI [0.12-1.64]), as compared to the Indo-Oceanic (‘ancient’) reference group. Patients infected with isolates belonging to the East-African Indian lineage (‘modern’) did not respond significantly different from patients infected with isolates belonging to the Indo-Oceanic lineage for any of the variables tested (Table 5, right model).

When comparing the largest family of isolates within each of the lineages defined by Gagneux *et al.*[22] LAM11_ZWE (‘modern’) induced a higher level of CRP (β = 24.4, *P* = 0.05; 95% CI [0.24-48.63]) and neutrophil count (β = 0.9, *P* = 0.03; 95% CI [0.09-1.70]) as compared to patients infected with isolates belonging to the ‘ancient’ EAI5 family reference group (Table 6). The level of AGP was also elevated among the patients infected with LAM11-ZWE isolates, however, this increase was not statistically significant (β = 0.4, *P* = 0.06; [-0.01-0.73]), possibly due to a low sample size. Patients infected with isolates belonging to the ‘modern’ CAS_KILI did not respond differently from patients infected with isolates belonging to the ‘ancient’ EAI5 family (Table 6). Adjustment for bacterial load (the number of colonies) did not affect the estimates (data not shown), also we found no associations between being infected with a ‘modern’ lineage isolate and having diabetes, smoking, TB contact and the presence of a BCG scar.

**Table 6:** **Associations between infection by different *M. tuberculosis *** **sublineages (according to the SpolDB4**[23]**) and clinical manifestations**

**Groups**	**Reference group: EAI5 (n = 33)**					
	**CAS_KILI (n = 44)**			**LAM11_ZWE (n = 51)**		
**Variables**	**Regression coefficient**	**95% CI**	**P-value**	**Regression coefficient**	**95% CI**	**P-value**
Age (years)^1^	1.8	[-4.55 – 8.06]	0.58	-0.6	[-6.84 – 5.68]	0.86
Weight (kg/m^2^)^2^	-1.0	[-4.37 – 2.48]	0.59	-3.0	[-6.39 – 0.40]	0.08
BMI (kg/m^2^)^2^	-0.5	[-1.62 – 0.67]	0.41	-0.9	[-2.07 – 0.19]	0.10
CRP (mg/L)^2^	13.2	[-11.26 – 37.66]	0.29	24.4	[0.24 – 48.63]	**0.05**
AGP (g/L)^1*^	0.2	[-0.15 – 0.62]	0.23	0.4	[-0.01 – 0,73]	0.06
Neutrophil count (x 10^9^ cells/L)^2^	-0.1	[-0.93 – 0.69]	0.77	0.9	[0.09 – 1.70]	**0.03**
Lymphocyte count(x 10^9^ cells/L)^2^	0.4	[-0.02 – 0.91]	0.06	0.3	[-0.18 – 0.74]	0.23
CD4 count (cells/ul)^2^	38.8	[-91.88 – 169.46]	0.56	-13.0	[-142.54 – 116.53]	0.84
Hemoglobin (g/dl)^2^	0.1	[-0.90 – 1.12]	0.83	-0.4	[-1.40 – 0.60]	0.43
Fasting blood glucose (mmol/L)^2^	-0.1	[-0.52 – 0.30]	0.60	-0.3	[-0.68 – 0.14]	0.19
Diabetes^2^	1.3	[0.35 – 4.99]	0.68	1.1	[0.28 – 4.22]	0.89
Smoking^2^	1.4	[0.44 – 4.22]	0.60	1.3^¶^	[0.41 – 4.19]	0.64
TB contact^2^	1.1	[0.39 – 3.12]	0.85	1.3^¶^	[0.46 – 3.44]	0.66
BCG scar^2^	1.2	[0.40 – 3.51]	0.76	1.5^¶^	[0.51 – 4.60]	0.44

Results are based on a linear and logistic regression models adjusted for age, sex and HIV status.

^1^Adjusted for HIV status and gender.

^2^Adjusted for HIV status, age and gender.

^3^*P*-values <0.05 are in bold.

*HIV negative only.

^¶^Odds ratio.

BMI: Body Mass Index.

EAI: East-African Indian.

CRP: C-reactive protein.

AGP: Orosomucoid.

BCG: Bacillus Calmette-Guerrin.

## Discussion

Few studies [28,29] have reported on the frequency of circulating genotypes in Tanzania as determined by spoligotyping. The current study indicates that 4 *M. tuberculosis* genotypes are responsible for ~55% of all TB cases in Mwanza City, Tanzania.

The genetic biodiversity of *M. tuberculosis* for a particular area can be attributed to, amongst others, geography and demography, and the level of human migration, travels, immigration and trade [28]. In this study, two genotypes, ST21 and ST59, belonging to the CAS_KILI and LAM11_ZWE lineages, respectively, comprised 37% of the 248 isolates analyzed. The predominance of these two families in the northern part of Tanzania has been demonstrated previously [23,28] and may suggest a biological advantage for these lineages in this region. CAS_KILI and LAM11_ZWE lineages are considered to belong to the ‘modern’ (TbD1-) *M. tuberculosis* lineages and are regarded as more recently evolved than the more ‘ancient’ EAI5 lineage. Interestingly, this study shows that isolates belonging to the more ‘modern’ Euro-American lineage induced higher levels of the acute phase response and neutrophil counts. Combined with a reduction of the BMI in patients infected with ‘modern’ lineage isolates, this finding suggest that ‘modern’ lineages induce an increased level of acute phase responses than isolates belonging to the more ‘ancient’ Indo-Oceanic lineage. However, the data presented for this study are cross-sectional limiting any interpretation of causality. Still, we found that the most abundant lineage, the ‘modern' Euro-American, was associated with a higher acute phase response as well as a higher leukocyte count. The high prevalence combined with more severe clinical manifestations indicate that the ‘modern' Euro-American lineage may be more virulent and, thus, more susceptible to transmission in this population compared to the ‘ancient’ and the ‘modern' East-African Indian lineages. It has previously been shown that more recent and evolutionary successful isolates of the Beijing family of strains elicit a lower cytokine response than the Euro-American linage [30]. As stated above; an organism’s virulence factors, is described as the severity of disease caused by that organism, or the pathogens infectivity. Thus, the virulence and infectivity of an organism cannot be explained only by its ability to induce a strong host response, as other factors such as the dynamic interaction between the host and the pathogen plays an important role in determining the outcome of the infection. Furthermore, this supports our findings that there are differences among the ‘modern’ families with regards to the degree of inflammation they cause. When comparing the two most common ‘modern’ families identified for this study (CAS_KILI and LAM11_ZWE) to the most common ‘ancient’ family (EAI5), this study showed that among the ‘modern’ families, there are differences in the level of acute phase responses caused by the two families. Whereas the CAS_KILI isolates induced similar levels as the EAI5 reference isolates for all variables tested, the LAM11_ZWE isolates induced a higher acute phase response in addition to higher neutrophil counts. As the presence of a visible BCG scar has been shown to have an effect on the sputum conversion at 2 months post anti-TB treatment [17] a possible linkage between the lineage of the infecting isolate and the presence of a visible BCG scar was investigated. No such correlation was observed.

The relatively low diversity of different genotypes in Mwanza City (15.7%) observed for this study, as compared to Dar es Salaam [52% [29]], is suggestive of a high degree of ongoing transmission of the most frequent genotypes, indicating an evolutionary advantage of the most common lineages over the other lineages combined with optimal conditions for transmission, such as an urban setting with overcrowding and inadequate ventilation. The Beijing lineage, a highly virulent genotype which is associated with high transmission and a high level of multi-drug resistance [4], was found at a very low frequency in the Mwanza city. The frequency (1.2%) of isolates belonging to the Beijing lineage observed in this study is low compared to a previous study [28] performed on isolates collected from northern Tanzania where the Beijing lineage was reported to be the 5th most frequent genotype. The low number of Beijing lineage isolates may contribute towards the very low number of multidrug-resistant isolates observed for this study. Furthermore, the very high frequency (95%) of isolates being sensitive to the four first-line anti-TB drugs is suggestive of a well-functioning treatment strategy.

Another cause for concern was the high number (51%) of mixed *M. tuberculosis* sub-populations observed in the isolates which were selected for MIRU-VNTR analysis. The presence of mixed *M. tuberculosis* sub-population among isolates belonging to the ST53 lineage has been demonstrated previously [25] and may be explained by the cumulative presence of spacers in all strains present, creating a ‘false’ ST53 pattern [24,25,31]. Multiple infections by different strains of *M. tuberculosis* are more likely to occur in high TB-endemic settings where the infection pressure is high. The true implications of a high level of mixed infections in a population is not clear however, it has been shown previously [32] that a mixed infection may be responsible for a change in drug-resistance pattern during treatment, and possibly may accelerate the emergence of multi-drug resistant isolates. As described by Lazzarini *et al.*[24], some spoligotype patterns are more likely than others to be the result of a mixed infection. Thus, in order to find the true frequency of the most dominating strains in Mwanza, further characterization, by MIRU-VNTR, would need to be performed for a larger selection of isolates belonging to the most dominating genotypes.

## Conclusion

The findings from this study indicate that ‘modern’ lineage isolates appear more virulent generally inflicting higher severity of the disease than ‘ancient’ lineage isolates. The low diversity of genotypes may be explained by an evolutionary advantage of the most common lineages over the other lineages combined with optimal conditions for transmission, such as overcrowding and inadequate ventilation. Ultimately, this may lead to increased transmission and spread of the ‘modern’ lineages in this area.

## Competing interests

The authors declare that they have no competing interests.

## Authors’ contributions

RS: Performed experiments, analysis and drafted the manuscript. GP: Conducting the main study, recruitment and follow-up of patients NR: Study design and manuscript writing. DFJ: Study design, performed the statistical analysis and drafted the manuscript. HK: Analysis of patient samples. MFJ: Analysis of patient samples. KJ: Conducting the main study, recruitment and follow-up of patients. MGA: Analysis of patient samples. JC: Conducting the main study, recruitment and follow-up of patients. HF: Final approval of the version to be published. ABA: Final approval of the version to be published. HMSG: Study design, manuscript writing. All authors read and approved the final manuscript.

## Pre-publication history

The pre-publication history for this paper can be accessed here:

http://www.biomedcentral.com/1471-2334/14/309/prepub
